# CHIP as a therapeutic target for neurological diseases

**DOI:** 10.1038/s41419-020-02953-5

**Published:** 2020-09-09

**Authors:** Shuo Zhang, Zheng-wei Hu, Cheng-yuan Mao, Chang-he Shi, Yu-ming Xu

**Affiliations:** 1 Department of Neurology, The First Affiliated Hospital of Zhengzhou University, Zhengzhou University, 450000 Zhengzhou, Henan China; 2 Henan Key Laboratory of Cerebrovascular Diseases, The First Affiliated Hospital of Zhengzhou University, Zhengzhou University, 450000 Zhengzhou, Henan China; 3 Academy of Medical Sciences of Zhengzhou University Translational Medicine platform, 450052 Zhengzhou, Henan China

**Keywords:** Cerebrovascular disorders, Neurodegenerative diseases

## Abstract

Carboxy-terminus of Hsc70-interacting protein (CHIP) functions both as a molecular co-chaperone and ubiquitin E3 ligase playing a critical role in modulating the degradation of numerous chaperone-bound proteins. To date, it has been implicated in the regulation of numerous biological functions, including misfolded-protein refolding, autophagy, immunity, and necroptosis. Moreover, the ubiquitous expression of CHIP in the central nervous system suggests that it may be implicated in a wide range of functions in neurological diseases. Several recent studies of our laboratory and other groups have highlighted the beneficial role of CHIP in the pathogenesis of several neurological diseases. The objective of this review is to discuss the possible molecular mechanisms that contribute to the pathogenesis of neurological diseases in which CHIP has a pivotal role, such as stroke, intracerebral hemorrhage, Alzheimer’s disease, Parkinson’s disease, and polyglutamine diseases; furthermore, CHIP mutations could also cause neurodegenerative diseases. Based on the available literature, CHIP overexpression could serve as a promising therapeutic target for several neurological diseases.

## Facts

- CHIP has dual function both as co-chaperone and ubiquitin ligase.
- CHIP can participate in inspecting and facilitating the refolding of misfolded proteins; moreover, it can also promote the degradation of ubiquitin-tagged proteins via the ubiquitin-proteasome pathway.
- CHIP overexpression can function neuroprotectively in neurological diseases, suggesting that it may constitute a promising therapeutic strategy.
- CHIP mutations can lead to spinocerebellar ataxia autosomal recessive 16 (SCAR16) or spinocerebellar ataxia 48 (SCA48).

## Open Questions

- What is the relationship between the location of the mutations and changes in the biological function of CHIP within the clinical spectrum of SCAR16 or SCA48?
- Why can CHIP mutations lead to coexistence of autosomal recessive and dominant inheritance?
- How can the most effective and practical new CHIP-based drugs for neurological disease treatment be developed?

## Introduction

It is established that proteins fundamentally contribute to the maintenance of cellular function and that protein stability is a major mechanism underlying human disease, such as cancer and neurological diseases. The organism has developed meticulous mechanisms to monitor and maintain the health of its proteome, which comprise the protein quality control (PQC) system. The most important means employed by the PQC system is the network of molecular chaperones, which can inspect and facilitate the refolding of misfolded proteins; moreover, it can also promote the degradation of ubiquitin-tagged proteins via the ubiquitin-proteasome pathway^1,2^.

Carboxy-terminus of Hsc70-interacting protein (CHIP), also termed STUB1, is a 35 kDa protein with the dual function of co-chaperone and E3 ubiquitin ligase activity^3,4^. CHIP is ubiquitously expressed and, in particular, highly expressed in hypermetabolic tissues with rapid protein turnover such as in skeletal muscle, heart, and brain tissues^3^. Moreover, CHIP is an evolutionarily ancient protein with extensive conservation across species; for instance, human CHIP exhibits 98% amino acid similarity with mouse CHIP^3^. CHIP comprises triple tandem tetratricopeptide repeat (TPR) domains, which can mediate interactions with chaperones such as HSP70 and HSP90 in the N-terminal end, an U-box domain displaying E3 ubiquitin ligase activity in the C-terminal end, and a central coiled coil (CC) domain with a largely unknown function that may be required for dimerization^5^. Due to its unique structure, CHIP can combine with chaperones via the TPR domain and mediate the ubiquitination and degradation of various chaperone-bound proteins via its E3 ubiquitin ligase activity^6–8^. Hence, CHIP can act as a connecting link between molecular chaperones and proteasomes and is widely regarded as a vital player in the cellular PQC system.

In recent years, growing evidence has shown that CHIP can also regulate the activity of non-misfolded proteins, such as INSR^9^, RIPK3^10^, TFEB^11^, and PKA^12^; in these pathways, CHIP does not degrade toxic proteins and even does not require chaperone partners, suggesting that CHIP can modulate the pathological process of human diseases through multiple pathways. Furthermore, according to the studies of our team and those of other groups^13–15^, some but not all CHIP mutants destabilize the structure and show defects in CHIP chaperone binding, which suggests that some CHIP mutations may cause diseases by influencing the non-canonical CHIP functions.

**Fig. 1:**
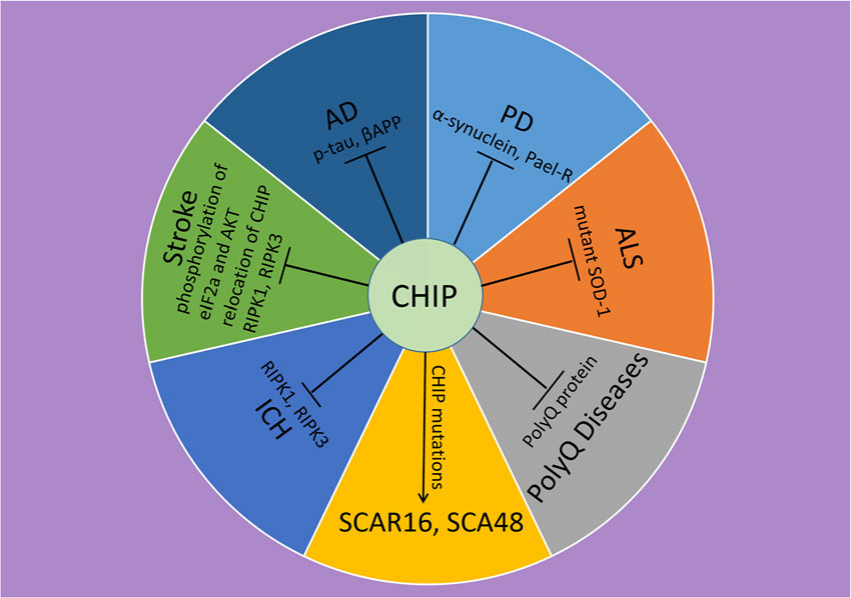
CHIP involves in various of nervous system diseases. Schematic diagram of the substrates of CHIP in the context of various neurological disorders. Please refer to the main text for details.

Emerging evidence indicates that CHIP is involved in multiple fundamental cellular processes relevant to the pathogenesis of neurological diseases. Herein, we will discuss in detail the available evidence of the physiological role of CHIP in the context of neurological diseases, such as intracerebral hemorrhage (ICH), ischemic stroke, Alzheimer’s disease (AD), Parkinson’s disease (PD), polyglutamine (PolyQ) diseases, and spinocerebellar ataxia autosomal recessive 16 (SCAR16) and spinocerebellar ataxia 48 (SCA48) caused by CHIP mutation (Fig. 1).

## Implication of CHIP in neurological diseases

### Intracerebral hemorrhage

ICH, which accounts for ~15% of all stroke-related events^16,17^, is a severe condition associated with high mortality and morbidity^18^. Mechanical damage caused by hematoma^19^ and secondary brain injury can cause neuronal death and neurological deficits^20^. Accumulating evidence has shown that strategies designed to limit neuron death after ICH are therapeutically valid to reduce hematoma expansion and improve outcomes. Recently, studies indicated that necroptosis^21–23^ is an important mechanism influencing brain injury after ICH and that inhibiting necroptosis can markedly ameliorate the outcomes^24,25^. As reported, CHIP can regulate necroptosis through mediating the degradation of RIPK3^10^, intimating the possible therapeutic function of CHIP after ICH.

Our team explored the role of CHIP following ICH^26^ and provided the first evidence that CHIP expression is upregulated after ICH in rat models. We constructed a CHIP overexpression rat model using AAVPHP.B viral particles, CHIP overexpression could remarkably reduce neurological scores and significantly decrease hemorrhagic-lesion volume after ICH^26^. Notably, CHIP overexpression observably decreased the number of necroptotic cells and the expression levels of RIPK1, RIPK3, and MLKL, three major regulators of necroptosis, around the hematomal region after ICH compared with those in the control groups. Furthermore, CHIP-deficient rats had significantly aggravated neurological impairments and markedly increased levels of RIPK1, RIPK3, and MLKL compared with wildtype (WT) rats; and these effects could be recovered through the reintroduction of CHIP. Moreover, CHIP overexpression ameliorated neuroinflammation in rats after ICH^26^.

However, the introduction of CHIP overexpression in our study was achieved through treatment with AAVPHP.B vectors prior to ICH induction, which is unattainable in clinical practice. Thus, it is necessary to develop effective and practical drugs that can rapidly induce CHIP overexpression. Moreover, the generation of neutralizing antibodies (NAbs) in peripheral circulation may be a major obstacle that impedes the repeated use of AAVPHP.B^27^. Therefore, establishing methods to circumvent NAbs should be a critical crux in the use of AAVPHP.B.

### Ischemic stroke

Stroke is a devastating disease threatening ~30 million humans annually worldwide, and ~80% of cases of stroke are of the ischemic type^28^. In addition to immediate neuron death after stroke, neuronal loss also occurs in the tissues surrounding the affected area due to multiple destructive mechanisms, such as excitotoxicity, necroptosis^29^, and inflammation. Therefore, therapeutic strategies that can rescue damaged neurons are very important, but approaches that can alleviate neurological impairment are limited. Hence, developing new therapeutic agents is urgently needed. Recent studies have demonstrated that CHIP participates in the pathological process of ischemic stroke; interestingly, the implicated mechanisms vary^30–34^. Anderson et al. reported that CHIP expression is upregulated in the postmortem brain tissues of patients after stroke^31^, implying that CHIP may play an important role in the pathological mechanism after stroke and may be a potential therapeutic target for neuronal injury.

In our study, CHIP protected N2a cells following oxygen glucose deprivation (OGD) through inhibiting necroptosis^30^. In detail, we detected upregulation of RIPK1, RIPK3, and MLKL in N2a cells exposed to OGD accompanied by increased cell death, and Nec-1, a RIPK1 inhibitor, could reverse these effects^30^. These results suggested that necroptosis was activated under OGD challenging. Moreover, anisomycin can reduce OGD-induced necroptosis through upregulation of CHIP expression in N2a cells and primary hippocampal neurons. As expected, CHIP overexpression could reduce the RIPK3 level by post-translational regulation under OGD-challenging. Moreover, while other functions of anisomycin may contribute to the benefit of OGD, the role of CHIP may be much more important^30^. Neither the TPR nor the U-box domain of CHIP alone possesses the protective function of inhibiting necroptosis, suggesting that both the co-chaperone and ubiquitin E3 ligase functions of CHIP are necessary for anti-necroptosis following OGD^30^. Similarly, Cabral-Miranda et al. reported that CHIP overexpression protected against hippocampal neuronal death through decreasing the phosphorylation of eIF2a and AKT in experimental brain ischemia^34^. Conversely, another study reported that CHIP overexpression exerted harmful effects and that downregulation of CHIP led to improved survival following OGD^33^; this distinction could likely result from the use of dissociated cell cultures, as Cabral-Miranda et al. suspected in their study^34^.

The relocation of CHIP subcellularly also plays an important role in ischemia^31^. In a cell model, the nuclear levels of CHIP increased in response to OGD, which can significantly delayed cell death, suggesting that there is an intimate relationship between CHIP nuclear localization and neuronal survival^31^. Furthermore, in an OGD neuronal model, CHIP and PINK1 expressions increased after stress, and CHIP was observed to relocate from cytosolic and perinuclear sites to the mitochondria following OGD^32^. Given that PINK1 changes synchronously with CHIP after OGD, we speculate that the re-localization of CHIP may be promoted by PINK1 in a similar manner to PINK1 recruitment of parkin, a E3 ligase^35–37^. Furthermore, CHIP-deficient neurons have lower cell viability compared to WT ones following OGD, which underscores the important role of CHIP in maintaining neuronal survival^32^.

As previously described, although the CHIP-related mechanisms in neuronal degradation or neuronal death vary, these studies have suggested that CHIP could be a promising therapeutic target to prevent brain injury after ischemic stroke.

### Parkinson’s disease

PD is the second most common neurodegenerative disorder and affects ~2% of the population over 60 years old. Although the pathogenesis of PD remains unclear, α-Synuclein (α-Syn), a major component of Lewy bodies, has long been associated with PD. α-Syn can form prefibrillar and fibrillar cellular aggregates, and these oligomeric assemblies can mediate α-Syn neurotoxicity^38,39^. Therefore, reducing α-Syn oligomerization or clearing existing a-Syn oligomers may provide a potential therapeutic target^38,39^. Shin et al. reported that CHIP plays a role in α-Syn aggregation and degradation^40^. They showed that CHIP co-localizes with α-Syn and Hsp70 in Lewy bodies and in α-Syn inclusions, and CHIP overexpression reduces α-Syn aggregation and increases α-Syn degradation. Moreover, CHIP can mediate α-Syn degradation by two discrete mechanisms, i.e., the TPR domain is critical for proteasomal degradation, but the U-box domain directs α-Syn toward the lysosomal degradation pathway^40^. Moreover, CHIP preferentially recognizes and mediates degradation of toxic oligomeric forms of α-Syn^41^, and that CHIP is an E3 ubiquitin ligase of α-Syn^42^. These results suggest that CHIP may be a target for PD treatment.

Although most cases of PD are sporadic, a Mendelian pattern of inheritance has also been recognized, such as cases of leucine-rich repeat kinase-2 (*LRRK2*)^43^, PTEN-induced putative kinase 1 *(PINK1)*^44^, and *Parkin*^45^. Mutation of *LRRK2* is the most frequent genetic cause of familial PD and has also been identified in individuals with sporadic PD^46^. Studies reported that CHIP binds, ubiquitinates, and promotes the degradation of LRRK2^47,48^. Hsp90 can attenuate CHIP-mediated LRRK2 degradation, and this can be blocked by a Hsp90 inhibitor^47,48^. Moreover, there are some variants that are not located in the central ROC-COR-kinase triple domain, including G2385R^49^. Rudenko et al. reported that G2385R LRRK2 has decreased kinase activity and affinity to the LRRK2 interactor^50^. Furthermore, G2385R LRRK2 has a lower steady state and can increase LRRK2 protein turnover compared to WT LRRK2^51^. CHIP can directly recognize different regions of LRRK2, including the WD40 domain where G2385R is located, and G2385R LRRK2 has a higher affinity for CHIP compared to WT LRRK2^51^. CHIP overexpression decreased both G2385R mutant and WT LRRK2, while CHIP knockdown had the opposite effect and induced neuronal death^51^. These results suggest that CHIP may be a potentially valid candidate for the treatment of LRRK2-related PD.

Mutation of *Parkin* is also a genetic cause of PD^45^. Parkin is a protein with a ubiquitin-like domain, and two RING finger motifs^52^. Pael receptor (Pael-R) is a substrate of parkin. Accumulation of Pael-R in the endoplasmic reticulum (ER) of dopaminergic neurons can induce ER stress, eventually leading to neurodegeneration^53^. CHIP, Hsp70, parkin, and Pael-R formed a complex both in vitro and in vivo^54^. The expression of CHIP in the complex was increased under ER stress. CHIP enhanced the dissociation of Hsp70 from parkin and Pael-R, then accelerating Pael-R ubiquitination mediated by parkin^54^. Moreover, CHIP enhanced parkin-mediated ubiquitination of Pael-R, and CHIP strengthened the ability of parkin to inhibit neuronal death induced by Pael-R^54^. Taken together, these data indicate that CHIP positively regulates parkin E3 activity and might be an excellent therapeutic target for the treatment of parkin-related PD.

*PINK1* is the second most frequent cause of autosomal recessive PD. Via its kinase activity, PINK1 regulates mitochondrial quality control and neuronal cell survival. CHIP is a novel ubiquitin E3 ligase that targets PINK1, promoting its ubiquitination and degradation, but this effect was not seen with mutants CHIP-H260Q and CHIP-ΔU^55^. CHIP overexpression suppressed PINK1 mutant phenotypes in flies, such as locomotion defects and loss of dopaminergic neurons^56^. Moreover, CHIP, but not its ligase-dead mutants, could rescue mitochondrial defects in PINK1 mutants^56^, suggesting that E3 ubiquitin ligase activity is required for CHIP to protect against mitochondrial dysfunction in PINK1-mutant flies.

### Alzheimer’s disease

AD is the most common neurodegenerative disease worldwide and is characterized by gradual onset of dementia, cognitive decline, even inability to live independently^57^. Although numerous causal hypotheses have been recently proposed, the presence of beta-amyloid (Aβ) deposits and hyperphosphorylated tau (p-tau) have gained most of the attention^58^. Thus, eliminating Aβ and p-tau may be promising therapeutic strategies.

Tau is a protein that plays a key role in the dynamics of microtubules. In the pathology of AD, tau becomes hyperphosphorylated, separates from the microtubules, misfolds, and aggregates into neurofibrillary tangles in the intracellular space, playing a critical role in AD^59,60^. Several studies have reported that CHIP can directly ubiquitinate and eliminate p-tau^61–66^. Moreover, CHIP strongly interacts with caspase cleavaged tau (tauΔC) compared to full-length tau (FL-tau), leading to increased ubiquitylation and rapid degradation of tauΔC^61^. Both the U-box and TPR domains are necessary for CHIP binding to tau; binding of Hsp70 to the TPR domain is necessary for reducing tau levels^61,63,64^. CHIP overexpression can also rescue the mitochondrial deficit caused by tau overexpression^63^. CHIP expression was increased in human brains with AD and tauopathy mouse models^67^, and CHIP deficiency could induce increase of insoluble tau^67^. Furthermore, tau expression was upregulated in the brain of CHIP-T246M rat model^13^, implying that tauopathy may be importantly implicated in the pathogenesis of CHIP mutation-related diseases. In an AD mouse model, overexpressing CHIP could reduce tau phosphorylation^68^. There results indicate that CHIP plays a critical role in mediated tau and p-tau degradation, and CHIP overexpression may protect against p-tau aggregation and neurofibrillary-tangle formation.

A large body of evidence supports that the accumulation of intracellular Aβ42, a proteolytic cleavage of amyloid precursor protein (APP) by β-site APP-cleaving enzyme 1 (BACE1), which plays a central role in AD pathogenesis by processing APP to Aβ, is a critical event in the pathogenic mechanism of AD, and reducing the accumulation of Aβ can mitigate the progression of AD^69–71^. Several studies have suggested that CHIP can act to lessen the toxicity of Aβ42^72–74^, and CHIP overexpression can directly interact with holo-βAPP and stabilize steady-state holo-βAPP levels^73^. In addition, a complex with several HSPs and CHIP can hasten the degradation of Aβ42 levels and protect against toxicity in neurons^73^. CHIP overexpression can decrease BACE1 levels by promoting its ubiquitination and degradation, thus reducing APP processing to Aβ^74^, and both the U-box and TPR domains are essential for ubiquitination and degradation of BACE1^74^. Furthermore, CHIP can also negatively regulate BACE1 through the regulation of p53-mediated transrepression^74^.

Moreover, emerging evidence has suggested that Aβ and tau may be mechanistically linked^75,76^, and exploration of the possible mechanistic links between Aβ and tau pathology may greatly aid the understanding of AD. One of the molecular mechanisms underlying the effects of Aβ on tau is mediated by CHIP^77^. Aβ42 plays a major role in the mechanism of tau pathology; reducing Aβ42 levels or preventing Aβ42 accumulation markedly delays the onset of tau pathology^77^. Moreover, Aβ42, not full-length APP, can reduce CHIP levels, which is sufficient to interfere with tau turnover and degradation, thereby facilitating the buildup of tau aggregates. More importantly, Aβ42-induced tau pathology can be rescued by restoring CHIP levels^77^. These results suggest CHIP as a valid therapeutic target, and increasing its activity may reduce Aβ42-induced tau pathology.

Several small-molecule agonists that can upregulate CHIP expression have been reported recently (Table 1)^30,68,78,79^. Sulforaphane, a herbal isothiocyanate draw from cruciferous vegetables, can clear the accumulation of Aβ and tau and then ameliorate learning and memory defaults in 3×Tg-AD mice through upregulating CHIP expression in the cortex and hippocampus^68^. Moreover, several traditional Chinese medications can also upregulate CHIP expression in the cerebral cortex and hippocampus of AD disease models, highlighting the role of CHIP in AD^80,81^. These results indicate that small-molecule agonists of CHIP such as sulforaphane may constitute promising drugs that could be used for the treatment of neurological diseases.

**Table 1:** Small-molecule agonists that can induce CHIP overexpression.

#	Small-molecule agonists	Disease model	References
1	Sulforaphane	3×Tg-AD mouse model	^68^
2	Anisomycin	Oxygen-glucose deprivation (OGD) cell model	^30^
3	Peptidoglycan (PGN)	RAW264.7 cells, peritoneal macrophage isolated from WT and TLR2 KO mice	^78^
4	2-(4-hydroxy-3-methoxyphenyl)-benzothiazole (YL-109)	MDA-MB-231 cells (breast cancer)	^79^

### Amyotrophic lateral sclerosis

Amyotrophic lateral sclerosis (ALS) is a late-onset fatal neurodegenerative disease selectively affecting motor neurons and other neuronal cells in the spinal cord, brainstem, and cortex, leading to severe disability and eventually death. The common pathological features of ALS include the accumulation of misfolded protein inclusions in motor neurons and other neurons in the cortex and other neuroanatomical regions. Approximately 10% of ALS cases are inherited familial cases (fALS), and genetic mutations in SOD1 are shared by 20% of patients with fALS^82,83^. Lines of evidence have indicated that the formation of aggregates is a common feature due to the misfolding of mutant SOD1 in over 100 SOD1 mutants^84^, and that mutant SOD1 is conjugated to a multi-ubiquitin chain and degraded in a chaperone-dependent manner, which suggests that UPS may play a critical role in the pathological mechanism of ALS.

CHIP can promote the degradation of mutant SOD1 but not of WT SOD1 at the proteasome^85^. Interestingly, CHIP does not directly target mutant SOD1, which differs from the mechanism of other CHIP substrates^85^. Moreover, CHIP immunoreactivity and high Hsc70-immunoreactivity are observed in inclusions of motor neurons of an ALS mouse model that contains SOD1 mutations, denoting the important role of the Hsp/Hsc70-CHIP machinery in the degradation of mutant SOD1^85^.

Dorfin is the first identified E3 protein that can specifically ubiquitylate mutant SOD1 and attenuate the cytotoxicity of mutant SOD1 in ALS cell models^86^. However, dorfin’s neuroprotective effect has been shown to be modest in dorfin/mutant SOD1 double transgenic mice^87^; this limited effect might be due to its short half-life^88^. Several studies have reported that engineered chimera E3s can degrade certain substrates with high efficiency^89–91^; Ishigaki et al. engineered a series of dorfin-CHIP chimeric proteins combined with the hydrophobic region of dorfin and the U-box domain of CHIP^87^. Some engineered proteins such as dorfin-CHIP^D, J, and L^ have longer half-lives and stronger binding activity than WT dorfin. Moreover, dorfin-CHIP^L^ presents stronger E3 activity when bound to mutant SOD1 compared to dorfin or CHIP alone, and dorfin-CHIP^L^ showed a greater therapeutic effect against mutant SOD1. It is noteworthy that dorfin-CHIP^L^ specifically ubiquitylates mutant SOD1 but not WT SOD1, just as dorfin^87^. Dorfin can be found in both fALS and sALS; and proteasomal abnormalities also occur in sALS. Thus, the neuroprotective function of dorfin-CHIP^L^ could render it an appropriate therapeutic candidate to be tested in clinical trials^87^. Furthermore, developing new chimera E3 ligases such as dorfin-CHIP^L^ may be a promising therapeutic concept for ALS and other neurodegenerative disorders.

### PolyQ diseases

PolyQ diseases are a group of inherited neurodegenerative diseases caused by a genetic mutation of the cytosine–adenine–guanine (CAG) triplet repeat expansion, which encodes for polyQ tracts in the causative proteins^92–94^. To date, nine disorders have been reported, including spinal and bulbar muscular atrophy (SBMA), Huntington’s disease (HD), several spinocerebellar ataxias (SCA1, SCA2, SCA3, SCA6, SCA7, and SCA17), and dentatorubral pallidoluysian atrophy (DRPLA)^95^. A common pathological feature of polyQ diseases is the degeneration of neurons in the brain. Misfolding and aggregation of the expanded polyQ proteins constitute the most upstream events in the most common pathogenic cascade of polyQ diseases; thus, approaches targeting misfolding and aggregation of expanded polyQ proteins are likely therapeutically important.

Several studies have reported that CHIP plays a critical role in regulating the degradation of polyQ^96–102^. CHIP can reduce the aggregation and increase the solubility of mutant huntingtin, which depends on the function of the TPR domain, and thus rescued inclusion formation and toxicity in disease models of HD^99^. Furthermore, CHIP deficiency can exacerbate and accelerate neuronal dysfunction and phenotype in an HD model^99^. Together, these results identified the beneficial effects of CHIP in HD, strengthening its potential as a therapeutic target for polyQ diseases. CHIP can interact with expanded huntingtin or ataxin-3, and that CHIP overexpression increased the ubiquitination and rate of degradation of expanded huntingtin or ataxin-3^100^. In a SCA3 mouse model, CHIP deficiency accelerated the disease and markedly increased the level of ataxin-3 micro-aggregates^100^. Moreover, CHIP overexpression suppressed the aggregation and neuronal death mediated by expanded polyQ proteins, and this function was more prominent when CHIP was overexpressed along with Hsc70^100^.

The role of CHIP in SCA1 has also been explored. CHIP and ataxin-1 directly interact and co-localize in nuclear inclusions (NIs) both in cell-model and patient postmortem neurons^98^. CHIP promotes the ubiquitination of expanded ataxin-1, and this effect involves the TPR domain^98^, Hsp70 can increase CHIP-mediated ubiquitination of ataxin-1^98^. Interestingly, CHIP also interacts with and ubiquitinates unexpanded ataxin-1^98^. Moreover, CHIP overexpression decreased the protein steady-state levels of both expanded and unexpanded ataxin-1 and suppressed their toxicity in a drosophila SCA1 model^98^. It is worth noting that the effect of CHIP is impaired by the mutation of Ser776 of ataxin-1 whose phosphorylation is critical for ataxin-1 aggregation^101^, suggesting that the role of CHIP in polyQ-protein aggregation greatly depends on the context of full-length polyQ proteins.

In addition, the role of CHIP has also been studied in SBMA. CHIP overexpression can improve motor disorders and inhibited nuclear accumulation of mutant AR in an SBMA animal model^97^. Moreover, mutant AR was preferentially degraded over WT AR under CHIP overexpression^97^, suggesting that mutant AR is more sensitive to CHIP than WT AR. These results demonstrate that CHIP overexpression mitigates SBMA phenotypes by reducing mutant AR by enhancing its degradation. Thus, we conclude that CHIP is a vital factor in response to misfolded polyQ proteins and represents a potential therapeutic target for polyQ diseases.

### Neurological diseases caused by CHIP mutations

Recently, there have been emerging reports of diseases caused by CHIP mutations. Gordon Holmes syndrome (GHS) is a rare neurodegenerative disease characterized by ataxia and hypogonadism^103^. In 2014, our team reported the first GHS family in China mainland and identified the third GHS-related pathogenic gene, i.e., CHIP with p.Thr246Met mutation in the U-box domain, which abolishes the ubiquitin ligase activity of CHIP^104^. Interestingly, the first and second pathogenicity genes of GHS, E3 ligase RNF216 and deubiquitinase OTUD4, also suggest that ubiquitination plays an essential role in the pathophysiology of GHS, further highlighting the role of deficiency of ubiquitin ligase activity in the pathogenesis of GHS^105^. Moreover, we found that CHIP knockout (CHIP^−/−^) mice also showed ataxia, hypogonadism, and cognitive impairment, closely reflecting the clinical manifestations of GHS patients. Histological examination of the CHIP^−/−^ mice cerebellum revealed cellular loss throughout the cerebellum specifically in the Purkinje cells compared with WT mice^104^. This strong similarity between our GHS patients and the CHIP^−/−^ rodent model suggests a vital role of CHIP in maintaining cerebellar function and the reproductive–endocrine axis^104^.

In further study, we found that CHIP p.Thr246Met mutation disrupted the structure of the U-box and promoted the formation of soluble oligomers, while still maintaining chaperone-interaction activity, i.e., interactions between CHIP and known interactors were not affected^13^. However, CHIP-T246M could change solubility and increase turnover. Moreover, we prepared mouse and rat models with the CHIP-T246M mutation, and both showed decreased expression of CHIP in the brain and cerebellum and decreased steady-state protein expression^13^. The CHIP-T246M rodent animal models recapitulated the key features of GHS^13^, which support our previous findings that CHIP plays a critical role in cerebellar maintenance. It is especially worth mentioning that some of the behavioral deficits in the CHIP-T246M rodent models were not shared by CHIP^-/-^ mice, implying that disease-causing mutations in CHIP and total loss of CHIP are not functionally equivalent; we suspect that while CHIP-T246M no longer functions as an E3 ubiquitin ligase, other CHIP functions remain intact^13^.

**Fig. 2:**
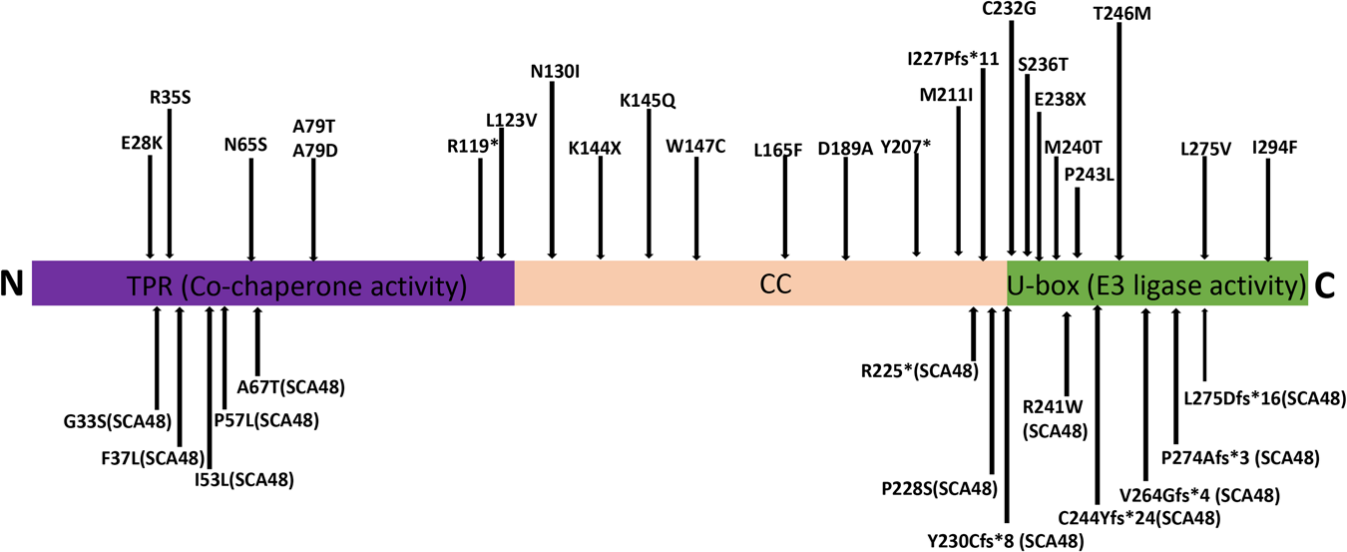
CHIP protein domains are diagramed. The locations (arrows) of the various mutations and respective nucleic acid and amino acid changes associated with SCAR16 and SCA48 are indicated in Table 2.

To date, more than 20 new pedigrees have been reported worldwide, and the autosomal recessive cerebellar ataxia caused by CHIP mutation was classified as SCAR16^104,106–116^ (Fig. 2 and Table 2). The pathogenic gene sites in CHIP of these patients were distributed in the U-box and TPR domains and even in the CC domain, suggesting that all three domains are indispensable in maintaining CHIP function. The clinical symptoms of SCAR16 are complex; in addition to early-onset progressive cerebellar ataxia, cerebellar atrophy, dementia, and hypogonadism, the patients also present with hyperkinetic movement disorder^114^ and significant pyramidal tract damage^113,114^, and in some patients, ataxia was not the first symptom^114^. Some patients with CHIP mutation also show action-verb impairment^117^. These findings imply that the phenotype of SCAR16 is not limited to ataxia syndrome, but rather involves almost the entire central nervous system. These results also show that CHIP not only plays a vital role in maintaining the cerebellum function but is also critically implicated in preserving the function of other areas of the brain.

**Table 2:** A summary of the various mutations and respective nucleic acid and amino acid changes associated with SCAR16 and SCA48.

#	Genotype	Amino Acid	Inheritance Mode	Disease	References
1	c.737C>T211	p.Thr246Met	AR^a^	SCAR16	^104^
2	c.612+1G>C, c.823C>G^b^	p.Leu275Val	AR	SCAR16	^106^
3	c.433A>C; c.687_690delCTAC^b^	p.Lys145Gln; p.Asn230Cysfs*8	AR	SCAR16	^107^
4	c.194A>G	p.Asn65Ser	AR	SCAR16	^116^
	c.82G>A; c.430A>T^b^	p.Glu28Lys; p.Lys144Ter			
5	c.433A>C; 721C>T^b^	p.Lys145Gln; Arg241Trp	AR	SCAR16	^108^
	c.433A>C; 694T>G^b^	p. Lys145Gln; Cys232Gly			
6	c.358+1G>A; c.566A>C^b^	p.Asp189Ala	AR	SCAR16	^109^
7	c.633G>A; c.712G>T^b^	p.Met211Ile; p.Glu238Ter	AR	SCAR16	^115^
8	c.389AT; c.441GT^b^	p.Asn130Ile; p.Trp147Cys	AR	SCAR16	^110^
	c.621CG; c.707GC^b^	p.Tyr207*; p.Ser236Thr			
	c.493CT	p.Leu165Phe			
9	c.103C>A; c.678_679del^b^	p.Arg35Ser; p.Ile227Profs*11	AR	SCAR16	^111^
10	c.367C>G,	p.Leu123Val,	AR	SCAR16	^112^
	c.719T>C,	p.Met240Thr,			
	c.235G>A; c.236C>A^b^	p.Ala79Thr; p.Ala79Asp			
11	c.*240T>C	Alteration the polyadenylation signal from AATAAA to AACAAA	AR	SCAR16	^113^
12	c.355C>T; c.880A>T^b^	p.Arg119*; p.Ile294Phe	AR	SCAR16	^114^
	c.433A>C; c.728C>T^b^	p.Lys145Gln; p.Pro243Leu			
13	c.823_824delCT	p.Leu275Aspfs*16	AD^c^	SCA48	^119,122^
14	c.731_732delGC	p.Cys244Tyrfs*24	AD	SCA48	^123^
15	c.158T>C	p.Ile53Thr	AD	SCA48	^124^
	c.111C>G	p.Phe37Leu			
16	c.97G>A,	p.Gly33Ser,	AD	SCA48	^125^
	c.682C>T	p.Pro228Ser			
17	c.170C>T, c.199G>A,	p.Pro57Leu, p.Ala67Thr,	AD	SCA48	^126^
	c.721C>T, c.673C>T,	p.Arg241Trp, p.Arg225*,			
	c.791_792delTG,	p.Val264Glyfs*4,			
	c.818_819dupGC,	p.Pro274Alafs*3,			
	c.689_692delACCT,	p.Tyr230Cysfs*9,			
	c.823_824delCT	p.Leu275Aspfs*16			

^a^Autosomal recessive.

^b^Compound heterozygous mutation.

^c^Autosomal dominant.

Consistent with our results^13^, studies of other CHIP mutations also found that some SCAR16 mutations destabilize CHIP, causing defects in CHIP function, including decreased interactions with chaperones, diminished substrate ubiquitination, increased formation of soluble oligomers, and reduced steady-state levels^14^. Astonishingly, two mutants, N65S and L123V, located in the TPR domain can also decrease the formation of free ubiquitin chains, implying that the co-chaperone function of the TPR domain may be important for activating the ability of E3 ligase activity^15^. However, it remains unclear whether there is a relationship between the location of the mutation and changes in the biological function of the corresponding mutated protein with the clinical spectrum of SCAR16. Madrigal et al. reported that the mutations located in the U-box domain strongly induce loss of CHIP function and are intensely related to cognitive impairment in patients with SCAR16^118^; TPR and CC mutations mildly affect CHIP function and are associated with an increased tendon reflex. They also reported that inhibiting the interaction between mutant CHIP and HSP70 can lead to later age of onset and less severe ataxia^118^.

In 2018, researchers reported a family with autosomal dominant cerebellar ataxia caused by a frameshift heterozygous STUB1 pathogenic variant, c.823_824delCT STUB1 (p.L275Dfs*16), which is distinct from SCAR16^119^. The same phenomena also occurred to *SPTBN2*, causing both SCA5 and SCAR15 due to dominant and recessive mutations, respectively^120,121^. The researchers named this disease SCA48^119,122–126^ (Fig. 2 and Table 2). The earliest and most prominent clinical manifestation of the patients was cognitive impairment, and cerebellar ataxia appeared years later^119^. Moreover, p.L275Dfs*16 STUB1 heterozygous carriers show no pyramidal signs or seizures, which can often be seen in patients with SCAR16, and extra-cerebellar symptoms can only be observed at the end stage^119^. Furthermore, c.823.824delCT of STUB1 leads to selective atrophy in cognitive- and emotion-related cerebellar areas that precedes the appearance of ataxia by years before motor-related cerebellar areas become involved^119^. It is worth noting that patients with SCA48 can also show cognitive impairment and motor cerebellar signs^119^. These results suggest that CHIP is a critical element in maintaining cognitive cerebellar function, but the mechanism requires further research.

Recently, two other SCA48 pedigrees respectively caused by p.Gly33Ser and p.Pro228Ser in CHIP were reported, and the phenotype of the patients appeared more complex^125^. In these two families with SCA48, the patients presented adult-onset ataxia associated with cognitive impairment and psychiatric disorders and a combination of movement disorders that have also been described in SCAR16 such as parkinsonism and dystonia; they also showed cerebellar atrophy and a widespread degeneration pattern in areas including the cerebellum, basal ganglia, and cerebral cortex^125^. The researchers also found a characteristic T2-weighted hyperintense signal extending from the dentate nuclei bilaterally to the middle cerebellar peduncles, which may be a useful imaging biomarker of SCA48^125^. Nowadays, patients with SCA48 with new mutations continue to be reported and their clinical features are more complex, including hypogonadism and extrapyramidal features^122^, which have also been observed in patients with SCAR16. Moreover, the extrapolated frequency in Lieto’s reference cohort reached 3.4% overall and ~23% among familial cases; the lack of evidence of founder mutations suggests that SCA48 may not be uncommon among the SCAs worldwide^126^. In these SCA48 families, p.Gly33Ser occurred in the TRP domain, whereas p.Pro228Ser and p.L275Dfs*16 were located in the ubiquitin ligase region, similar to findings pertaining to more than 10 SCAR16 families, and it can be seen that both protein regions are critical in maintaining CHIP function.

Several studies regarding the function of mutated CHIP have reported that some STUB1 mutations mediate disease by affecting the CHIP E3 ubiquitin ligase interactions and function through modification of its oligomeric states and structural stability, which indicates that change in structure may lead to gain- or loss-of-function and thus cause disease. However, we should also note that numerous mutations have limited effects on the structure and ubiquitination activity of CHIP, suggesting that these mutations may affect CHIP function though other mechanisms^13–15,118^. The most intensively studied mutation site in vitro and in vivo was T246M, and these studies could serve as grounds for further research of other CHIP mutation sites.

According to these studies, an interesting question remains regarding why CHIP mutations can lead to the coexistence of autosomal recessive and dominant inheritance. It is evident that carrier parents in SCAR16 families are healthy, although they bear mutation protein products in their brains. The type and location of the mutations cannot explain this phenomenon, as missense, frameshift, or nonsense mutations occurring in SCAR16 are scattered in the U-box and TPR domains and even in the adjacent regions without hotspots. Therefore, the pathogenetic mechanisms of SCA48 and its relationship with SCAR16 should be further explored.

Besides the use of rodents or cells, other model systems, such as worms and drosophilas, are also widely used in studying the role of CHIP in neurodegenerative diseases^9,56,98^. For example, Tawo et al. reported a competitive relationship between proteostasis and longevity regulation through CHIP-assisted proteolysis^9^, and almost all the experiments were performed using worms and flies, denoting that worms and flies can serve as good model animals for exploring the role of CHIP.

As almost all CHIP mutations in SCAR16 and SCA48 are associated with abnormal function, gene therapy delivering functional gene copies of CHIP may be the obvious beneficial solution; alternatively, gene-editing approaches or antisense oligonucleotide therapy may also prove to be effective therapeutic avenues.

## Conclusions

In this review, we highlighted the neuroprotective role of CHIP in neurological diseases such as stroke, AD, and PD, and diseases caused by CHIP mutations. These results suggest that CHIP overexpression could be a potential therapeutic target. However, almost all these studies introduced CHIP overexpression via gene transfection, which may be of limited clinical use, and thus further studies are required to determine the most effective and practical regimens for CHIP treatment in neurological diseases. Furthermore, the combination of whole-exome sequencing analysis with traditional linkage analysis is a fruitful approach that can further extend the phenotypical spectrum of rare Mendelian disorders.
